# The identity, distribution, and impacts of non-native apple snails in the continental United States

**DOI:** 10.1186/1471-2148-7-97

**Published:** 2007-06-26

**Authors:** Timothy A Rawlings, Kenneth A Hayes, Robert H Cowie, Timothy M Collins

**Affiliations:** 1 Department of Biological Sciences, HLS Bldg, Rm. 318B, Florida International University, University Park, Miami, Florida 33199, USA; 2 Center for Conservation Research and Training, University of Hawaii, 3050 Maile Way, Gilmore 408, Honolulu, Hawaii 96822, USA; 3 Department of Zoology, University of Hawaii, Honolulu, Hawaii 96822, USA; 4 Department of Biological Sciences, Cape Breton University, PO Box 5300, 1250 Grand Lake Road, Nova Scotia, B1P 6L2, Canada

## Abstract

**Background:**

Since the mid 1990s populations of non-native apple snails (Ampullariidae) have been discovered with increasing frequency in the continental United States. Given the dramatic effects that introduced apple snails have had on both natural habitats and agricultural areas in Southeast Asia, their introduction to the mainland U.S. is cause for concern. We combine phylogenetic analyses of mtDNA sequences with examination of introduced populations and museum collections to clarify the identities, introduced distributions, geographical origins, and introduction histories of apple snails.

**Results:**

Based on sampling to date, we conclude there are five species of non-native apple snails in the continental U.S. Most significantly, we recognize three species within what has been called the channeled apple snail: *Pomacea canaliculata *(California and Arizona), *Pomacea insularum*, (Florida, Texas, and Georgia) and *Pomacea haustrum *(Florida). The first established populations of *P. haustrum *were discovered in the late 1970s in Palm Beach County Florida, and have not spread appreciably in 30 years. In contrast, populations of *P. insularum *were established in Texas by 1989, in Florida by the mid to late 1990s, and in Georgia by 2005, and this species continues to spread rapidly. Most introduced *P. insularum *haplotypes are a close match to haplotypes from the Río Uruguay near Buenos Aires, indicating cold tolerance, with the potential to spread from Florida, Georgia, and Texas through Louisiana, Alabama, Mississippi, and South Carolina. *Pomacea canaliculata *populations were first discovered in California in 1997. Haplotypes of introduced *P. canaliculata *match native-range haplotypes from near Buenos Aires, Argentina, also indicating cold tolerance and the potential to establish farther north.

**Conclusion:**

The term "channeled apple snail" is descriptive of a morphology found in many apple snail species. It does not identify a single species or a monophyletic group. Clarifying species identifications permits a more accurate assessment of introduction histories and distributions, and provides a very different picture of the tempo and pattern of invasions than was inferred when the three species with channeled sutures were considered one. Matching introduced and native-range haplotypes suggests the potential for range expansion, with implications for native aquatic ecosystems and species, agriculture, and human health.

## Background

Members of the freshwater gastropod family Ampullariidae, known as apple snails, have an impressive track record as invasive species. Species in three genera, *Pila*, *Pomacea*, and *Marisa*, have demonstrated a tenacious ability to survive and spread rapidly in the freshwater habitats into which they have been introduced [e.g. [1-3]]. Of these, members of the genus *Pomacea*, which is native to South and Central America, parts of the Caribbean, and the southeastern U.S., have become widely established in many areas within Southeast Asia, Sri Lanka, Guam, Hawaii, Papua New Guinea, the Dominican Republic, parts of the mainland U.S., and possibly Australia [4-6]. Many of these introductions have resulted from the escape or release of snails from plant or animal aquaculture operations [e.g. [2]]. Other introductions have probably resulted from release of snails acquired through the pet trade [e.g. [3]], a common introduction pathway for alien species [7].

Within the continental U.S., there is only one native apple snail, *Pomacea paludosa*. This species is widely distributed across the wetlands of the Florida peninsula and extends into Florida's panhandle as far west as the Choctawhatchee River, and into warm springs in Georgia [8]. A population also exists in Alabama, where it was introduced in 1953 [9]. Over the past 50 years, however, *Marisa cornuarietis *and several species of *Pomacea *have invaded wetlands and waterways of the mainland U.S., and established populations of these non-native ampullariids are now present in six states: Alabama, Arizona, California, Florida, Georgia, and Texas [10]. Since their initial appearance in the 1950s, the introductions of non-native apple snails largely occurred unimpeded and without much apparent concern [but see [11]]. This changed dramatically in the 1990s, however, with the appearance and rapid spread of channeled apple snails, thought to be *Pomacea canaliculata*, in rice-growing areas of Texas.

Channeled apple snails represent a major risk to native wetland ecosystems and agriculture. The name "channeled apple snail" was first coined as an Anglicization of the scientific name of the presumed single species *Pomacea canaliculata*, with reference to the distinct sutural channel between adjacent shell whorls. Nevertheless, this is a feature of many species of *Pomacea*, including *Pomacea insularum*, and *Pomacea haustrum*, and it has not been clear whether channeled apple snails are a single species or even a monophyletic group [10,12]. In Southeast Asia, *Pomacea canaliculata *and *Pomacea insularum *have become devastating agricultural pests, especially of rice [4-6], raising concern for U.S. rice growing regions. In addition, these species have the potential to alter native freshwater habitats significantly, causing shifts in ecosystem state and function [13]. In response to these concerns, the U.S. Department of Agriculture began requiring permits for importation or interstate movement of aquatic snails in 2006, specifically targeting apple snails with the hope of limiting their spread in the U.S.

Despite mounting concern, the identity of non-native ampullariids in the continental U.S., their provenance, current distributions, and means of introduction and spread remain poorly understood. Difficulty in species identification is largely a consequence of the overall conservative external morphology of the group combined with phenotypic plasticity of *Pomacea *species. Few rigorous morphometric studies have been carried out on the family [14-16], and the degree of overlap and variability in shell morphology makes delimitation of many species based on shell shape unreliable. This difficulty is complicated further by the fact that the aquaculture industry and pet trade have not been especially concerned with the species-level taxonomy of the snails introduced [e.g. [17]].

The first known ampullariid introduction into the continental U.S. was of *Marisa cornuarietis*, discovered in South Florida in 1957 [11] and probably released through the pet trade. The introduction history of other ampullariids is less clear. *Pomacea bridgesii *was first reported in Florida by Clench in 1966 [18]. In 1978, snails identified as *Pomacea canaliculata *were collected from Palm Beach County, Florida [8]. Since then, apple snails with channeled sutures have been reported in other Florida counties, and in Alabama, Arizona, California, Georgia, and Texas, with many of these reports since about 2000.

A critical first step in management and control of alien ampullariids is to ascertain what species are present, their distributions, where they came from, and when the populations became established. By "established" we mean the presence of self-sustaining populations in natural bodies of water or man-made canals and lakes, excluding aquaculture facilities and other locations where the snails may have been maintained in artificial environments. Correct identification of species and their origins permits access to published information on their biology and ecology in their native habitats, which could be useful in understanding the tolerances, potential non-native ranges, and possible impacts as invasive species. Determining the timing of establishment helps us to understand the epidemiology of the invasion, that is, the rate and dynamics of the invasion process. Here, we use molecular phylogenetic analyses to clarify the identities, origins, and distributions of *Pomacea *species within the continental U.S. We supplement this with information from museum collections and illustrate shells and egg masses of each species. We then attempt to determine the time and place of initial establishment of each species, and discuss potential impacts to native ecosystems, agriculture, and human health.

## Results and Discussion

### Species-Level Phylogenetic Analysis

**Figure 1:**
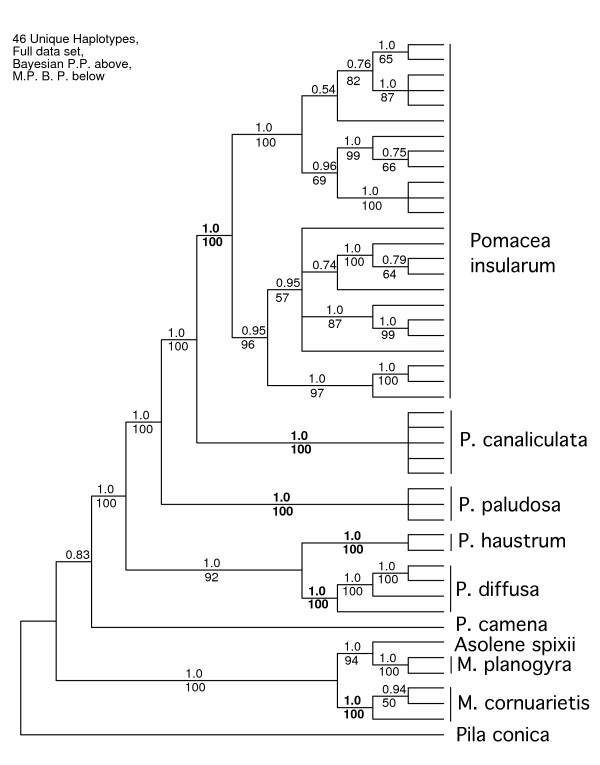
**Phylogenetic relationships among 46 unique mitochondrial haplotypes from 95 individual apple snails for which complete sequences were generated**. Analyses were based on combined *12S*-*16S *(~1,900 bp) and *COI *(~658 bp) datasets including native-range and introduced apple snail samples. Locality information is provided in subsequent figures and Additional file 1. Bayesian posterior probabilities (P.P.) are listed above branches, maximum parsimony (M.P.) bootstrap percentages (B.P.) below. The support values for clades containing non-native apple snails as well as a clade of *Pomacea paludosa *are shown in boldface.

All introduced ampullariids in the continental U.S. fell into five well-supported clades based on analysis of the 46 unique mtDNA haplotypes (Fig. 1). Based on comparison with native-range samples and type material, and knowledge of type localities (see below), these clades represented three apple snail species with channeled sutures (*Pomacea insularum*, *Pomacea canaliculata*, and *Pomacea haustrum*), and, also, *Pomacea diffusa *and *Marisa cornuarietis*. *Pomacea canaliculata *was strongly supported as sister to *P. insularum*, with *Pomacea paludosa *robustly supported as sister to these two species.

Discrete *P. haustrum *and *P. diffusa *clades formed a clade that was sister to the *P. insularum*, *P. canaliculata*, and *P. paludosa *clade. The position of *Pomacea camena *was not as robustly supported and varied among gene regions analyzed. *Marisa cornuarietis *haplotypes formed a well-supported monophyletic clade that was sister to a clade of *Marisa planogyra *and *Asolene spixii*, indicating that *Marisa *is not monophyletic.

Of the three apple snail species with channeled sutures in the continental U.S., *Pomacea canaliculata *occurred only in California and Arizona, *P. insularum *occurred in Texas, Georgia, and Florida, and *P. haustrum *was restricted to Florida.

Our results indicated that: 1) South American *Pomacea *were not monophyletic and 2) apple snails with channeled sutures were not monophyletic. This latter result is perhaps not surprising. The shells of many *Pomacea *species are similar in overall shape; unadorned spirally coiled tubes with overlapping whorls that do not depart appreciably from the axis of coiling. Given these general constraints, simple changes in the rate of shell whorl expansion and translation down the axis of coiling will affect the degree of overlap of shell whorls, and consequently the degree of channelling of the suture. The degree of channeling may also be affected by changes in the cross-sectional shape of the whorl. Convergence in channeled shells is thus not unexpected among the fifty or so species within the genus *Pomacea*.

### Taxonomy, Source Populations, and Introduced Distributions

Our results indicate that five species of non-native ampullariids have been introduced to the continental U.S. Below, we assess their probable geographic origins, the timing of their initial introductions, their spread, and current distribution. We conclude with an assessment of the risks to environments, native species, agriculture, and human health that these species pose.

#### Pomacea haustrum (Reeve, 1856)

**Figure 2:**
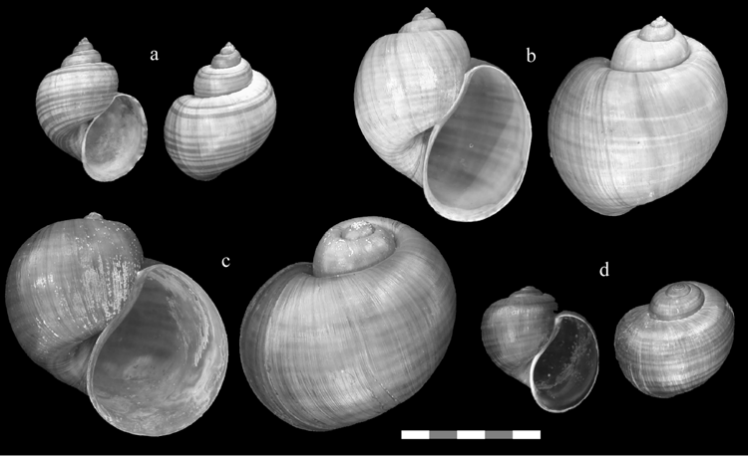
**Shells of introduced and native *Pomacea *collected in Florida**. Apertural and abapertural views of the same specimens: a. *Pomacea diffusa*, b. *Pomacea haustrum*, c. *Pomacea insularum*, d. *Pomacea paludosa *(the native Florida apple snail). Scale Bar: 5 cm.

**Figure 3:**
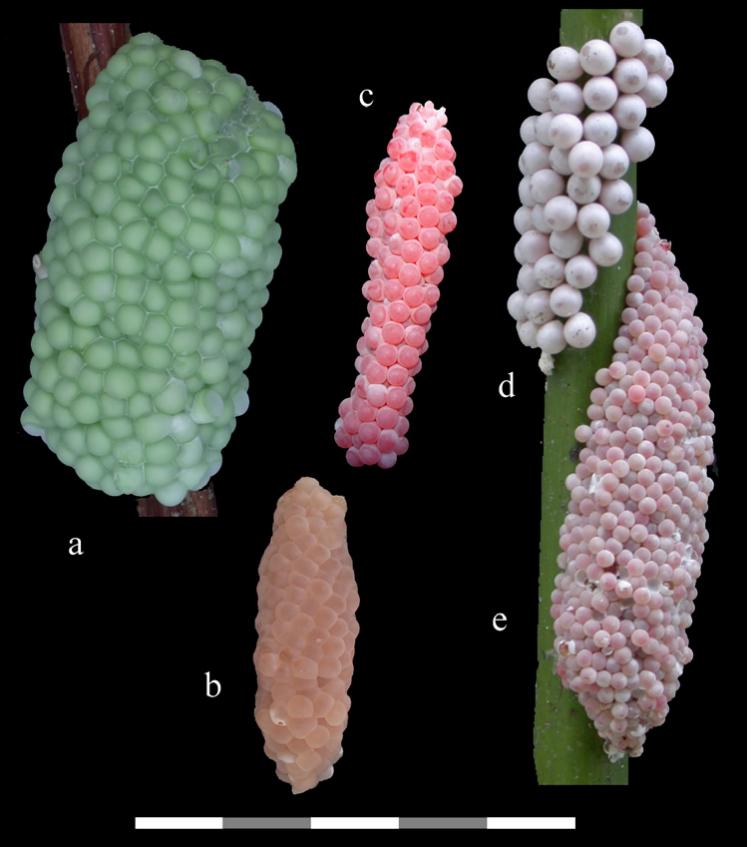
**Egg masses of introduced and native *Pomacea *in the continental U.S**. a. *P. haustrum*, b. *P. diffusa*, c. *P. canaliculata*, d. *P. paludosa*, e. *P. insularum*. Scale Bar:5 cm.

**Figure 4:**
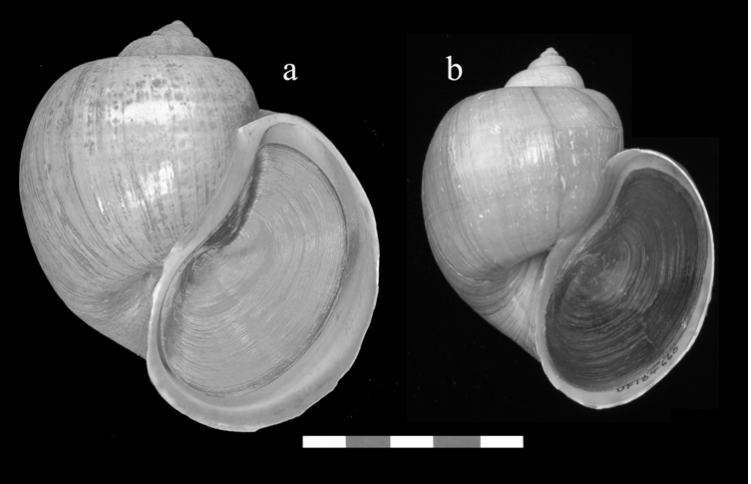
**Morphological correspondence between the possible syntype of *Pomacea haustrum* and *Pomacea* specimens collected in Palm Beach, Florida**. a. Possible syntype of *Pomacea haustrum *(BMNH 20020660), b. Specimen originally identified as *Pomacea canaliculata *collected in 1978 in Florida (FLMNH 184660), which we believe to be *Pomacea haustrum*. Scale Bar: 5 cm.

*Pomacea haustrum *is a large species with a channeled suture (Fig. 2b) that is native to Brazil, Peru, and Bolivia [19]. It was considered a synonym of *Pomacea canaliculata *by Thompson [20] based on shell morphology, but tentatively retained as a separate species by Cowie and Thiengo [12], because of its reported production of green eggs. Populations sampled in Florida produce bright green egg masses consisting of individual eggs approximately 3–5 mm in size compressed into polygonal shapes, giving the egg mass an irregular honeycombed appearance (Fig. 3a). Since other *Pomacea *species also produce green eggs [4], this character is insufficient to verify this species as *P. haustrum*. Nevertheless, the Florida material corresponds closely in shell morphology with the possible syntype of *P. haustrum *in the Natural History Museum, London (Fig 2b, Fig. 4a, b).

It is difficult to identify even the general region of the type locality for this species. The type locality was given as the Río Marañón, Brazil [21], but this river is in Peru, joining with the Rio Ucayali above Iquitos to become the Rio Solimões, which in turn joins with the Rio Negro to become the Amazon River of Brazil. Some of the first Europeans to explore the region in the sixteenth century, however, referred to the Amazon River as El Río Marañón, and some nineteenth century maps refer to the entire Amazon as the Marañón [22]. It is likely that the Río Marañón of one collector was not the same as the Río Marañón of another.

**Figure 5:**
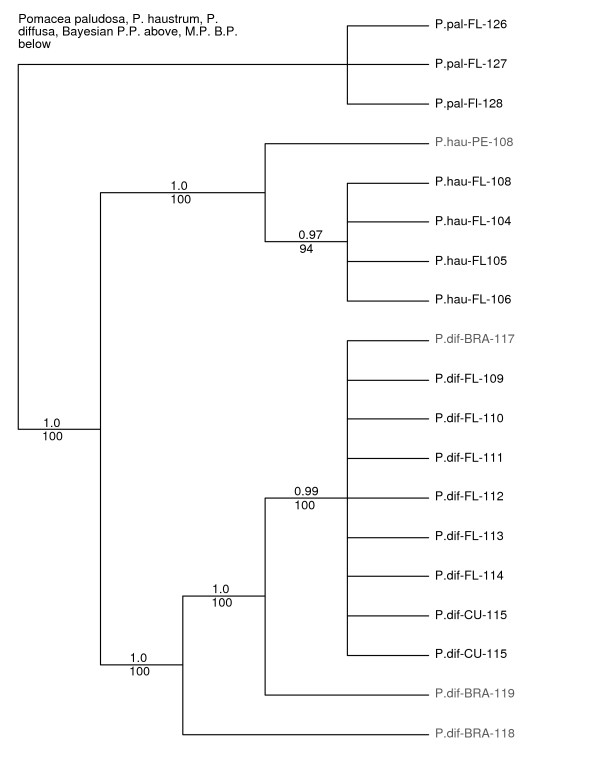
**Phylogenetic analysis within the *P. haustrum/diffusa *clade with *Pomacea paludosa *as the outgroup**. Analyses were based on combined *12S*-*16S *(~1,900 bp) and COI (~658 bp) datasets including native-range and introduced apple snail samples. Bayesian posterior probabilities are listed above branches, parsimony bootstrap percentages below. Abbreviations are: P.pal = *P.paludosa*; P.hau = *P.haustrum*; P.dif = *P.diffusa*; FL= Florida; BRA = Brazil; CU = Cuba. For specific locality information see Additional file 1.

We extracted DNA from a specimen identified as *P. haustrum *(FMNH 223530) from the Rio Ucayali in Peru, above its confluence with the Río Marañón. We were able to amplify an ~511 nucleotide portion of the 16S rRNA gene. This sequence grouped with specimens from Florida identified as *P. haustrum *in a well-supported clade (Fig. 5) and differed over this region by 1.4% (7 nucleotides). We conclude that the sum of current information supports the identification of this species as *Pomacea haustrum*, probably derived from populations in the Brazilian or Peruvian Amazon River system.

The only established populations of *P. haustrum *known by us in the U.S. are in Palm Beach County, Florida. As of June, 2006, adults and egg masses were present in the Loxahatchee National Wildlife Refuge and in canals and lakes of some surrounding communities (Collins and Rawlings, pers. obs.).

*Pomacea haustrum *was reported as established in Palm Beach County after 1989 [23], although a specimen of *P. haustrum *was collected there as early as 1983 [10]. We believe, however, that specimens collected in the 1970s and 1980s attributed to *P. canaliculata *(see below) are *P. haustrum*. For example, the earliest Florida specimen identified as *P. canaliculata *in the Florida Museum of Natural History (FLMNH 184660) was collected in 1978 from Palm Beach County and corresponds closely to the possible syntype of *P. haustrum *(Fig. 4). Also, an egg mass attributed to *P. canaliculata *(FLMNH 142245, collected in 1989) has traces of green color, the irregular honeycombed appearance, and the egg size of *P. haustrum*. Therefore, *P. haustrum *was probably introduced and successfully established by the late 1970s, but has failed to expand its range appreciably since then.

#### Pomacea canaliculata (Lamarck, 1822)

The apple snails with channeled sutures introduced into the continental U.S. have been identified as *Pomacea canaliculata *[e.g. [20,24]] or referred to as part of the *Pomacea canaliculata *complex or group [10]. Cowie and Thiengo [12] listed the native distribution of *Pomacea canaliculata *as Argentina, Bolivia, Paraguay, Uruguay, and Brazil. Nevertheless, Cazzaniga [25] suggested that all *canaliculata*-like ampullariids may constitute a single, very variable species, distributed throughout most of tropical and warm-temperate South America. The difficulties in discriminating *Pomacea canaliculata *and similar species, particularly *Pomacea insularum *(see below), have been noted [26,27] and reflect not only this poor understanding of its native range but also its considerable intraspecific morphological variability [15,28,29]. The type locality of *P. canaliculata *(rivières de la Guadeloupe) is ambiguous, but may refer to Lago Guadeloupe, Argentina [25].

**Figure 6:**
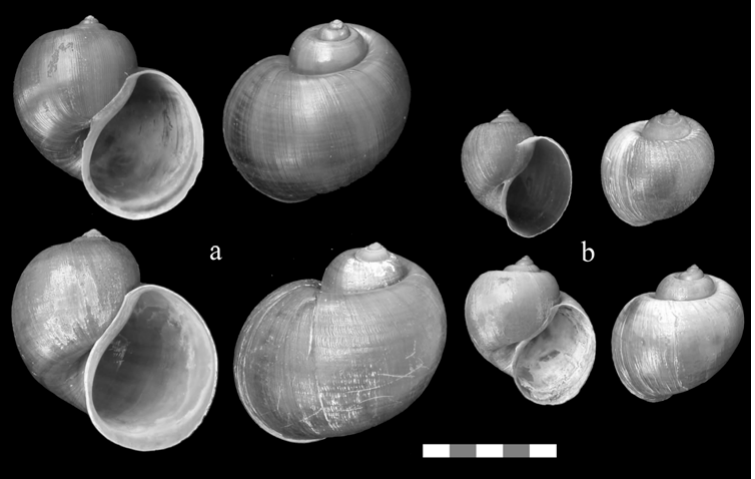
**Shells of *Pomacea insularum *and *Pomacea canaliculata *collected within the continental U.S**. Apertural and abapertural views of two specimens of: a. *Pomacea insularum *from Lake Tohopekaliga, Florida; b. *Pomacea canaliculata *from Mecca, California. Scale Bar: 5 cm.

Introduced populations of *Pomacea canaliculata *are genetically distinct from those of *P. insularum*, based on mtDNA sequences (Fig. 1), but these species have similar shell morphologies (Fig. 6). The egg masses of *P. canaliculata*, although bright reddish pink, like those of *P. insularum*, contain fewer but larger eggs (Fig. 3c) [27]. Clutches of *Pomacea insularum *often exceed 1,000 eggs (Fig. 3e). The variability in clutch size reported for *P. canaliculata *[4,30] may reflect inclusion of data for other species, particularly *P. insularum*. There is some preliminary evidence that these species are reproductively isolated [31].

**Figure 7:**
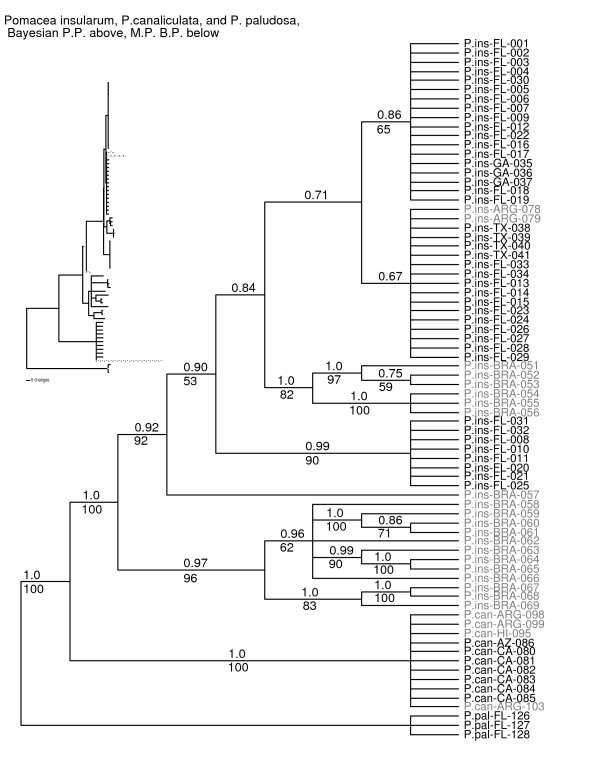
**Phylogenetic relationships within the *P. insularum/canaliculata *clade using *Pomacea paludosa *as the outgroup**. Analyses were based on combined *12S*-*16S *(~1,900 bp) and *COI *(~658 bp) datasets including native range and introduced apple snail samples. Bayesian posterior probabilities are listed above branches, bootstrap proportions below. Taxon labels of specimens collected from outside the continental U.S. are shown in grey font. Relative branch lengths are illustrated in the phylogram shown in the inset. Abbreviations are: P.ins = *P.insularum*; P.can = *P.canaliculata*; P.pal = *P.paludosa*; FL= Florida; GA = Georgia; TX = Texas; BRA = Brazil; ARG = Argentina; HI = Hawaii; AZ = Arizona; CA = California. For specific locality information refer see Additional file 1.

In our phylogenetic analyses *P. canaliculata *and *P. insularum *are reciprocally monophyletic sister taxa (Fig. 7) with a mean haplotype difference of 6.30%, compared to 1.29% within *P. insularum*, and 0.02% within *P. canaliculata*. We found very little genetic variation in *P. canaliculata*. Over 2,405 aligned positions, the greatest pairwise difference was 2 nucleotides, and identical haplotypes of *P. canaliculata *were found in snails from two locations in California, Hawaii, and Buenos Aires. The Arizona sequence differed from the Argentinean one by a single nucleotide. The source of the introduced populations of *P. canaliculata *is therefore likely to be Argentina, specifically the Buenos Aires area [K.A. Hayes, R.C. Joshi, S.C. Thiengo, and R.H. Cowie, in prep.].

In the continental U.S., *P. canaliculata *is found only in Arizona and California, although we have not sampled the Alabama population of apple snails with channeled sutures. The East and West coast populations of apple snails with channeled sutures are therefore different species, and need to be treated as distinct management units. The match of haplotypes to Hawaiian samples suggests a possible pathway for its introduction to the western U.S., via the food trade.

*Pomacea canaliculata *was reported in Florida and Texas in the late 1970s, probably based on misidentification of *P. haustrum *in Florida and *P. insularum *in Texas. The earliest report of an established population of *P. canaliculata *in California is 1997 in Lake Miramar, north of San Diego [24]. The history of other populations in California and Arizona was reviewed recently [10].

#### Pomacea insularum (d'Orbigny, 1835)

The difficulties of discriminating *Pomacea insularum *from *P. canaliculata *have been reviewed above. *Pomacea insularum *is reported from Argentina, Brazil [12], and Bolivia [32] and it probably occurs in Uruguay and Paraguay.

The type locality is the Río Paraná, which joins the Río Uruguay just above Buenos Aires, forming the Río de la Plata. The area between the Paraná and the Uruguay is the Argentine province of Entre Ríos, the southern part of which is marshy, with channels connecting the Paraná and the Uruguay. We sampled a specimen of *P. insularum *(FLMNH 159738) from Concepción del Uruguay on the Uruguay, which is closely linked to the type locality. We amplified and sequenced ~1,900 nucleotides, which corresponded closely to sequences from several populations of introduced *P. insularum*, differing by only two nucleotides (0.1%).

**Figure 8:**
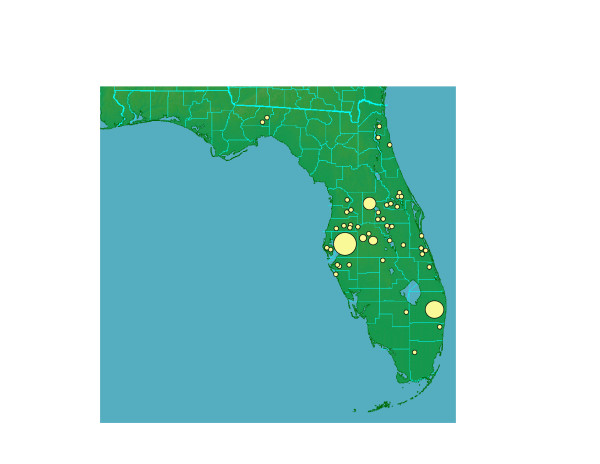
**Location of populations of *Pomacea insularum *in Florida**. In areas where many populations are in close proximity, a larger circle encompasses the area. Localities are based on our own observations/collections and a database compiled by Dana Denson, Florida Department of Environmental Protection, as of June 2006.

Based on our analyses to date, *P. insularum *is restricted to Texas, Florida, and Georgia. It has been recorded from a large number of localities in Florida (Fig. 8), the majority in the center of the state between Tampa and Orlando, but with other populations near major human population centers such as Jacksonville and Tallahassee. We have also found populations in ecologically sensitive areas including Everglades National Park and Loxahatchee National Wildlife Refuge.

*Pomacea insularum *has not been reported previously in the continental U.S. Specimens identified as *P. canaliculata*, collected in 1978 in Palm Beach County, are *P. haustrum *(above). When, then, did *P. insularum *become established in Florida? Winner [23] reported ampullariids with pink egg masses in Florida fish farms in 1991. Specimens collected in Hillsborough (FLMNH 261647) and Collier Counties (FLMNH 254749) in Florida in 1996 may be *P. insularum*. At present, the earliest genetically confirmed specimen that is from the location of an extant population was collected in 2002 in Lake Munson (FLMNH 298514). Many populations were discovered in Florida from 2004 to 2006. The establishment of *P. insularum*, misidentified as *P. canaliculata*, may be considerably more recent than has heretofore been thought, and its spread may therefore be much more rapid than has been appreciated.

Howells *et al*. [10] reviewed the distribution history of *P. insularum *(as "*Pomacea canaliculata*-group") in Texas. The first established population (identified as *P. canaliculata*) was discovered in 1989 in Houston (Harris County) [33]. Populations have since been documented in the surrounding counties of Brazoria, Galveston, Waller, Fort Bend, and Chambers [10].

In Georgia, *P. insularum *was first collected from the Alabaha River, in Blackshear (Pierce County) in February 2005. Other populations, some established, have since been discovered in ponds and streams of the Alabaha River, on St. Simon's Island (Glynn County) and at the mouth of the St. Marys River (Camden County) (Brett Albanese, pers. comm.).

**Figure 9:**
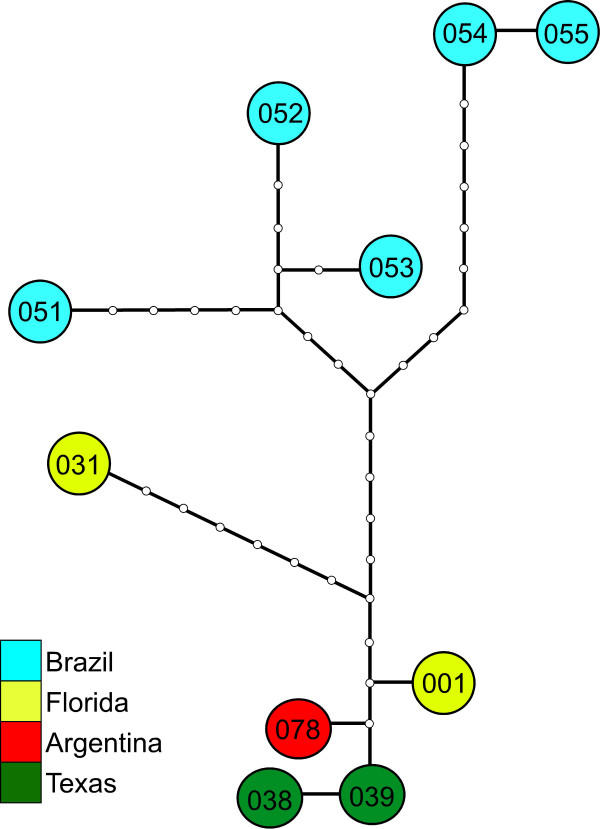
**Parsimony network within *Pomacea insularum*. **Statistical parsimony analysis was undertaken using TCS [version 1.20] on 48 *P. insularum *sequences. Shown are the 10 unique haplotypes listed by locality and identity code (see Figure 7; Additional file 1). For specific locality information refer to the corresponding numbered specimen of *P. insularum *in Additional file 1. Note that a native range haplotype of *P. insularum *from Argentina (ARG-078) is nested within introduced North American haplotypes.

We found considerable intraspecific variation and well-supported phylogenetic structure in *P. insularum *(Fig. 1), although missing data from a few museum specimens degraded clade support in the *P. insularum-P. canaliculata *analysis that encompassed all specimens (Fig. 7). The Georgia, Texas, and the majority of the Florida samples grouped with the Argentina samples from the Río Uruguay (Fig. 7). Statistical parsimony analysis showed that the Argentina haplotype was nested within the network of introduced haplotypes, further supporting its close relationship to introduced populations (Fig. 9). Although the majority of introduced snails sampled were most closely related to the Río Uruguay haplotype, a small number formed a distinct well-supported clade (Fig. 7), with weak support for its placement as sister to the clade of Argentinean haplotypes in the full haplotype analysis (Fig. 1). In the analysis of all *P. insularum *sampled, this clade was nested, although not robustly, between two clades made up of native-range samples from Brazil (Fig. 7). We do not know yet whether these two clades differ in ecologically relevant traits.

#### Pomacea diffusa Blume, 1957

*Pomacea diffusa *is known as the spike-topped apple snail, because of its relatively raised spire. It lacks a channeled suture, and overlaps in size with the native *P. paludosa *(Fig. 2a) [8]. The egg masses have an irregular honeycombed appearance, like those of *P. haustrum*, but are smaller and have a tan to salmon color (Fig. 3b), although the egg masses are white when freshly laid. *Pomacea diffusa *was originally described as a subspecies of *Pomacea bridgesii*. Pain [19] argued that *P. bridgesii bridgesii *was a larger form with a restricted range, with the smaller *P. bridgesii diffusa *being the common form throughout the Amazon Basin (Brazil, Peru, Bolivia). Cowie and Thiengo [12] suggested that the latter might deserve full species status, and the two taxa have been confirmed as distinct species by genetic analyses [[27], K.A. Hayes, R.C. Joshi, S.C. Thiengo and R.H. Cowie, in prep.].

The type locality of *Pomacea diffusa *is in the city of Santa Cruz, Bolivia, although the species is widespread throughout the Amazon Basin. Three samples from Belém, Brazil, formed a well-supported clade with non-native populations from Florida and Cuba (Fig. 5), exhibiting 0.4 to 1.8% sequence difference from the introduced populations.

Thompson [8] recorded this species (as *P. bridgesii*) in Florida in Monroe, Miami-Dade, Broward, Palm Beach, and Pinellas Counties. The FLMNH electronic database also lists samples from Alachua County, but records cited from the FLMNH database for Brevard County [e.g. [10]]) are in fact from Broward County. We have also collected this species in Hillsborough and Collier Counties.

*Pomacea diffusa *was first recorded in Florida (as *P. bridgesii*) by Clench [18]. The FLMNH has specimens collected in Palm Beach County in 1967 (FLMNH 20295) and Miami-Dade and Broward Counties in the early 1970s (FLMNH 22175, 222247). Howells *et al*. [10] reported its establishment in Mobile, Alabama in 2003.

#### Marisa cornuarietis (Linnaeus, 1758)

**Figure 10:**
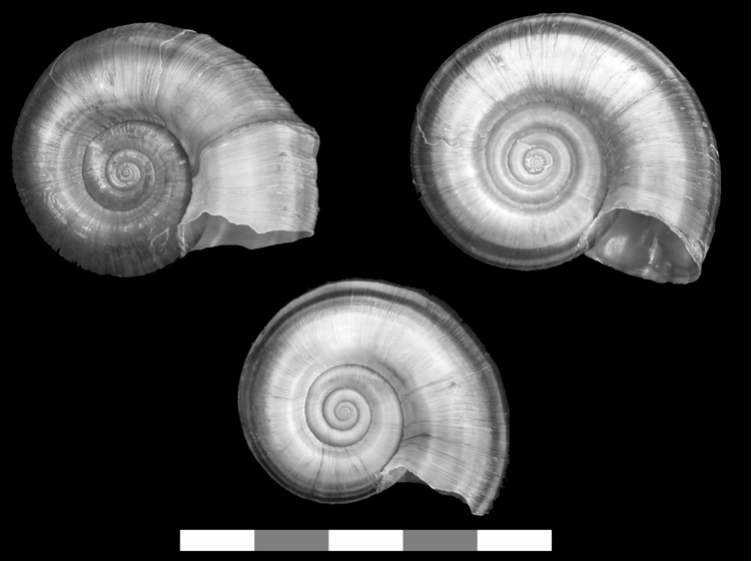
**Apical view of shells of *Marisa cornuarietis *collected in Florida**. Scale Bar: 5 cm.

*Marisa cornuarietis *is a distinctive planispiral ampullariid (Fig. 10). Its egg masses are unique among introduced ampullariids in being gelatinous throughout development and in being laid in water [34]. It is widespread in northern South America, although the type locality is unknown. There are currently two recognized species in the genus, *M. cornuarietis *and *M. planogyra *[12]. We included samples of both species in our analysis: non-native *M. cornuarietis *from the Caribbean island of Guadeloupe, and *M. planogyra *from its native Pantanal region of Brazil, near its type locality. We also included *Asolene spixii*, which clustered within the *Marisa *clade, thereby rendering *Marisa *paraphyletic. The introduced *Marisa *we sampled fell within a well-supported clade of *Marisa cornuarietis*.

*Marisa cornuarietis *was first discovered in Coral Gables, Florida, in 1957 [11]. It has spread to many other counties in southern Florida [[8], Rawlings and Collins, pers. obs.]. It was first found in Texas in 1983 [35] and has also been reported in California and Idaho [10]. Its potential ecological impacts have been reviewed recently [10].

### Potential Impacts of Non-native Pomacea

*Pomacea haustrum *is currently of relatively minor concern in the U.S., given its failure to spread beyond Palm Beach County after 30 years or more in Florida. It should be noted, however, that many species have maintained limited distributions, sometimes for decades, before becoming invasive [36]. *Pomacea diffusa *has generally been assumed also to pose little threat in the U.S. and it is the only apple snail for which interstate transport is permitted. This lack of concern may be unwarranted. The U.S. Department of Agriculture considered it (as *P. bridgesii*) to be innocuous [37], presumably based on a study that concluded that it feeds primarily on aufwuchs, not macrophytes [38]. The potential effects of *Pomacea diffusa *in natural habitats are unknown, but a conflicting study reports that, in addition to macrophytes, it will feed readily on animal carcasses, live worms, and the eggs of planorbid snails [39]. It may therefore have direct effects on both aquatic vegetation and native snails and compete for food with native scavengers such as crayfish, shrimp, and fish.

*Pomacea insularum *and *P. canaliculata *pose the greatest threat to agriculture and native wetland ecosystems in the U.S. One of the better predictors of the effect of an invasive species is the effect of the species or related species in other areas where it has been introduced [40]. The potential of *P. canaliculata *has been clearly demonstrated in Southeast Asia where its introduction into a tropical wetland ecosystem in Thailand resulted in dramatic changes in biodiversity and ecosystem functioning [13,41,42], and by its devastating effects on agriculture, especially wetland rice production [4-6]. Currently, the occurrence of *P. canaliculata *has been confirmed in California and Arizona in the continental U.S. However, it has the potential to spread into other areas, including the rice-growing parts of California, where it could cause serious damage.

Some of the ecological and agricultural impacts in Asia associated with *P. canaliculata *are almost certainly attributable to *Pomacea insularum*. This species is also widespread in the region [[27,43], K.A. Hayes, R.C. Joshi, S.C. Thiengo, and R.H. Cowie, in prep.; see below], but has not been explicitly acknowledged as a serious pest because of the confusion in identification of these two species, with most of the literature referring to *P. canaliculata. Pomacea insularum *may therefore be likely to have a significant impact on aquatic ecosystems and pose a threat to crops in the southeastern U.S. [10], particularly given the potential for it to spread through parts of Alabama, Mississippi, Louisiana and Texas.

The match of introduced haplotypes of *P. canaliculata *and *P. insularum *to native Argentinean samples from approximately 35°S suggests that the introduced populations of these species may be cold tolerant and capable of surviving occasional frosts. Moreover, *P. canaliculata *occurs as far as 38–39°S, and topography rather than climate may set the natural southern limit of this species [44,45]. The average minimum monthly temperature in Buenos Aires is 4–6 degrees Celsius (39–43 degrees Fahrenheit) from May to September, slightly lower than the average minimum winter monthly temperatures in Charleston, South Carolina. Consequently, *P. insularum *could potentially spread at least this far north on the Atlantic Coastal Plain, and through parts of Alabama, Mississippi, Louisiana, and Texas on the Gulf Coastal Plain. Similarly, *P. canaliculata *may be able to spread from its current introduced locations in California at least as far north as San Francisco. Ecological niche modeling based on the native and introduced range of these species [e.g. [46]] will permit a more refined estimate of potential introduced ranges. An important caveat here is that reports of the native ranges of these two species may be commingled.

**Figure 11:**
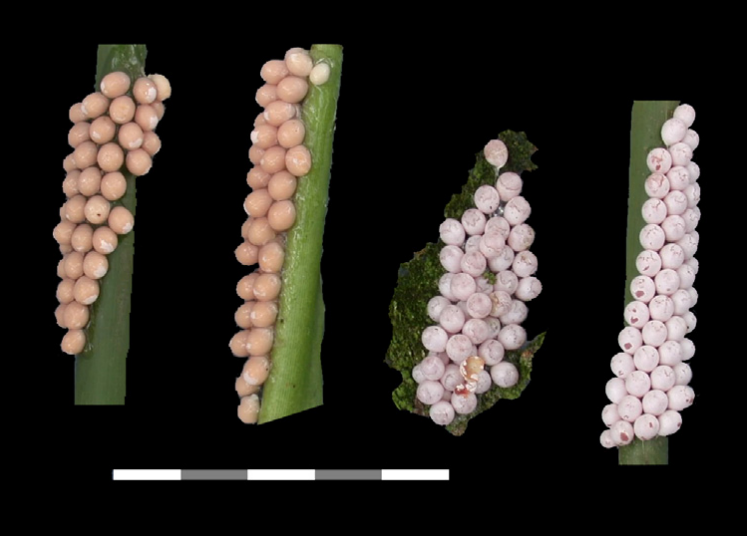
**Maturation of *Pomacea paludosa eggs***. Maturation from freshly laid salmon colored eggs in a thick mucus matrix (left), to the mature pinkish white eggs in calcified shells (right). Scale Bar: 5 cm.

*P. insularum *also poses threats for the native apple snail, *Pomacea paludosa*, and the species that rely on it for food. *Pomacea paludosa *is recognized by its size (40–70 mm height), low spire, absence of a channel at the suture, and distinctive egg masses [8]. Mature egg masses have an average of thirty round pale pink to white eggs averaging 4 mm in size in grape-like clusters [47] (Fig. 3d), although when freshly laid, the eggs are pale orange salmon colored and in a thick mucus matrix (Fig. 11). Those planning control measures aimed at non-native apple snails in Florida must ensure they have not inadvertently targeted the native apple snails or their eggs.

In addition to collateral damage from control of non-native species, "extinction by hybridization" [48] could result from hybridization of *P. paludosa *with non-native species, which seems possible given the close phylogenetic relationship of *P. insularum *and *P. paludosa*. There is also the possibility of competitive interactions between native and non-native snails [49]. Introduced *Pomacea *spp. have been reported to consume the eggs of both congenerics and other snails and invertebrates [39,50,51]. We have anecdotally noted a decline in abundance or the apparent disappearance of *P. paludosa *following introduction of *P. insularum *(Collins and Rawlings, pers. obs.). *Pomacea paludosa *is the primary food of the endangered Everglade Snail Kite [52], and an important food for other birds, including the limpkin, as well as fish, turtles, and alligators. At Lake Tohopekaliga (Collins and Rawlings, pers. obs.), limpkins are routinely taking *P. insularum *for food.

Finally, introduction of all non-native ampullariids is a concern because of the suite of associated parasites and their potential effects on apple snail predators and humans. We know nothing at present about the parasites of introduced ampullariids in the continental U.S. Ampullariids, including *Pomacea canaliculata*, are intermediate hosts of important vertebrate parasites [53], most notably nematodes in the genus *Parastrongylus *(=*Angiostrongylus). Parastrongylus cantonensis *causes eosinophilic meningoencephalitis [54], and *P. costaricensis *causes abdominal angiostrongyliasis in humans [55]. Transmission of *Parastrongylus *infections to mammalian hosts requires development to the L3 larval stage in an intermediate gastropod host. The spread of *Pomacea *species may therefore facilitate the spread of *Parastrongylus *species by completing the life cycle required to infect mammalian hosts. In Beijing, China, consumption of snails identified as *Pomacea canaliculata *resulted in 131 cases of human *Parastrongylus cantonensis *infection during a 4-month period [56]. *Parastrongylus cantonensis *and/or *P. costaricensis *are established in Florida and Louisiana [55,57-59], so the potential interaction of *Pomacea insularum *with *Parastrongylus *species is a concern.

## Conclusion

Clarification of species identifications, introduced ranges, and native ranges provides a more accurate picture of the tempo of apple snail invasions in the continental U.S., and suggests considerable potential for future range expansion. The situation is dynamic: during the time in which this paper was written, a new population of apple snails with channeled sutures has been discovered in New Orleans (S. Longman, pers. comm.), and preliminary genetic data (T. Collins and J. Bernatis, unpublished data) suggest that a population of *P. canaliculata *is present in north Florida. Considerable work remains to be done. We have sampled only about one quarter of the Florida populations of apple snails with channeled sutures, and the ranges of introduced ampullariids remain incompletely known. Other species may yet be discovered in introduced populations. At present, a combination of genetic data, morphological characters, and egg mass morphology is the best strategy for definitive identification. Finally, our genetic results are based on a single marker, although preliminary evidence from the nuclear histone 3 gene gives similar results [Hayes *et al*., in prep]. Higher resolution markers such as microsatellites may be useful in indicating the pathways by which non-native snails are spreading rapidly through parts of the U.S.

## Methods

### Sampling, DNA extraction, and sequencing

We sampled native *Pomacea paludosa *in Florida and non-native apple snails in Florida, Georgia, Texas, California, Arizona, Hawaii, Cuba, and Guadeloupe in the Lesser Antilles [see Additional file 1]. These included taxa identified as *Pomacea canaliculata *or *Pomacea canaliculata*-group, *Pomacea bridgesii*, *Pomacea haustrum*, *Marisa cornuarietis, Pila conica *and *Pila polita*. We also sampled native-range populations of *Pomacea canaliculata*-group snails, *Pomacea haustrum*, *Pomacea diffusa*, *Pomacea camena*, *Asolene spixii*, *Marisa planogyra*, and *Pila polita. Pila conica *was used as an outgroup for phylogenetic analyses [60,61]. Specimens were also obtained from the Bishop Museum (Honolulu, BPBM), Field Museum of Natural History (Chicago, FMNH), Florida Museum of Natural History (Gainesville, FLMNH), the Muséum National d'Histoire Naturelle (Paris, MNHN), and the Natural History Museum (London, BMNH) for both molecular and morphological comparisons.

DNA was isolated from ~50 mg of tissue using standard phenol/chloroform methods [62], or Qiagen's Dneasy extraction kit. The polymerase chain reaction (PCR) was used to amplify two portions of the mitochondrial genome. The first consisted of ~1,900 nucleotides spanning the 3'end of the *12S *rRNA, the intervening tRNA *valine *and the 5'end of the *16S *rRNA. This region was amplified in two overlapping portions using the primers 12Sai [5'-AAACTAGGATTAGATACCCTATTAT-3'; 63] with 16Srgast [5'-GCCATGATGCAAAAGGTAC-3'], and 12Sf [5'-GCACACATCGCCCGTCGCTCT-3'; reverse complement of 12Sb' in 63] with 16SbrH-alt [5'-CCGGTCTGAACTCAGATCATGT-3'; slight modification of 16Sbr in 63]. The second region was a ~658 nucleotide portion of the *cytochrome c oxidase *subunit I (*COI*) gene amplified using slightly degenerate versions of standard primers [64]. Reactions were performed in a MJ Research PTC-200 thermal cycler in 50 μl volumes with 1.5 mM MgCl_2_, each dNTP at 200 micromolar, 10–100 nanograms of genomic DNA, and 1X Promega buffer B, and 1 U of Taq polymerase (Promega, Madison, Wisconsin). PCR products were purified with a GeneClean III Kit (Bio 101, Carlsbad, California), and cycle-sequenced with Big Dye version 3.1 chemistry following the manufacturer's protocol (PE-ABI). Sequencing reactions were analyzed on an ABI Prism 3100 Genetic Analyzer. All samples were sequenced on both strands and sequences have been deposited in GenBank: rRNAs [GenBank: EF519073–EF519181, AY449500] and *COI *[GenBank: EF514942–EF515081].

COI sequences were generated for 141 snails. The *12S*-*16S *region was sequenced for representatives of each unique *COI *haplotype and locality combination, for a data set of 108 individuals. For some specimens, primarily museum specimens, amplification did not work for all gene regions, and different gene regions failed to amplify in different snails, resulting in snails with no sequence in common. This resulted in significant degradation of support values. We therefore selected the 95 individuals with both gene regions completely sequenced, representing 46 unique haplotypes, for the among-species phylogenetic analysis. Nevertheless, some individuals with a great deal of missing data were important for matching introduced haplotypes to native ranges, and were therefore used for within-species analyses and haplotype networks.

### Alignment and phylogenetic analyses

COI sequences were aligned at the amino-acid level and back aligned to nucleotides without indels in MacClade [65]. The rRNA sequences were aligned using T-Coffee [66] on the IGS server with default settings [67]. We tested alignment sensitivity by repeating phylogenetic analyses on a data set in which ambiguously aligned regions were removed by excluding positions in each direction until we came to nucleotides that were invariant in all taxa in the analysis [68].

We used maximum parsimony and Bayesian methods for phylogenetic analyses. Maximum parsimony bootstrap analyses were performed using PAUP* 4.0b10 [69] with equal weighting, uninformative characters excluded, and gaps treated as missing data. Three hundred bootstrap replicates with 10 random sequence additions per replicate (RSAs) and TBR branch swapping were performed using the heuristic search option. We used the Akaike Information Criterion (AIC) to select the most appropriate model of sequence evolution using Modeltest 3.07 [70]. Results indicated that the *12S*-*16S *sequence and the intervening tRNA sequence could be combined in one partition. The *COI *sequence was divided into 3 partitions by codon position. The parameters determined by Modeltest were used to specify the models of sequence evolution, and as priors for the Bayesian analyses, which were performed using MRBAYES 3.1.2 [71]. The prior distributions for the Bayesian analyses were as follows: topology uniform; branch lengths exponential with parameter = 10.0; gamma shape parameter exponential with parameter = 5; proportion invariant sites uniform over the interval (0, 1); and substitution rate matrix and base frequencies Dirichlet with starting values from Modeltest. We employed 4 Markov chains for 3–6 million generations and the partition-specific models described. Trees were sampled every 100th generation, discarding the first 33% of sampled trees as burn-in. We initiated two simultaneous runs from different random starting trees, and convergence was inferred when the average standard deviation of split frequencies approached 0.01, and the potential scale reduction factor for all parameters approached 1.0.

To explore further the relationships among introduced haplotypes of *P. insularum *from Florida, Texas, Georgia, and native-range populations, we performed a statistical parsimony analysis using TCS [version 1.20, 72]. We included 48 individuals based on the results of phylogenetic analyses (below). Some museum specimens did not amplify for all regions, resulting in significant missing data for a few individuals. Because missing data may cause problems related to calculated distances and collapsing of haplotypes in the program TCS, we eliminated characters with missing data or individuals with excessive missing data, and performed the TCS analysis based on 1781 nucleotides for which there are no missing data, with a parsimony limit of 90%.

## Authors' contributions

RC and KH generated the COI sequences, and contributed the majority of the native-range and the Hawaiian samples for genetic analyses. TC and TR generated the rRNA sequences and contributed the majority of the continental U.S. and museum samples for genetic analyses. TC and TR reviewed the relevant apple snail collections in the FMNH and FLMNH, and RC the holdings in the BPBM and MNHN. RC and TR examined the apple snail collections in BMNH. TR and TC carried out the alignments and phylogenetic analyses and drafted the manuscript. All authors participated in the design of the study and revision of the manuscript. All authors read and approved the final manuscript.

## Supplementary Material

Additional File 1 Species sampled in this study, GenBank accession numbers, collecting localities, and collectors. Click here for file
